# The Longitudinal Interplay Between Loneliness and Depressive Symptoms During Late Childhood: Cross-Lagged Panel Network Analyses

**DOI:** 10.3390/ejihpe16060078

**Published:** 2026-05-31

**Authors:** Paweł Grygiel, Sylwia Opozda-Suder, Roman Dolata

**Affiliations:** 1 Faculty of Philosophy, Jagiellonian University, Golebia 24, 31-007 Cracow, Poland; sylwia.opozda@uj.edu.pl; 2 Faculty of Education, University of Warsaw, Mokotowska 16/20, 00-561 Warsaw, Poland; rdolata@uw.edu.pl

**Keywords:** loneliness, depressive symptoms, prospective relations, late childhood, cross-lagged panel network

## Abstract

Background: Loneliness and depression are interrelated constructs that significantly impact adolescents’ mental health. Understanding their interplay, particularly at the symptom level, is critical for developing effective interventions. Objective: To examine longitudinal relationships between loneliness and depressive symptoms during late childhood, aiming to identify symptom-level interactions and directional effects. Participants and Setting: A total of 4333 children (*M*_age_ = 11.06, *SD* = 0.73; 50.8% girls) from the NLSY79 Children and Young Adults survey participated, with data collected over two years. Methods: A cross-lagged panel network (CLPN) model was employed to analyze symptom-level associations between loneliness and depressive symptoms. This approach combines network analysis and cross-lagged panel modeling, allowing for the estimation of both autoregressive effects (stability of symptoms over time) and cross-lagged effects (directional relationships between symptoms across time points). Results: The longitudinal network suggests the following: (1) a reciprocal link between loneliness and both sadness and parental pressure; (2) a forward effect of loneliness on anxiety and being busy; (3) the loneliness-reducing effect of prior happiness and loneliness-increasing effect of boredom. Conclusions: The findings highlight the complex interplay between loneliness and depressive symptoms, emphasizing reciprocal and unidirectional effects at the symptom level. These insights underscore the need for targeted, symptom-focused interventions to address loneliness and its impact on adolescent mental health.

## 1. Introduction

Loneliness and depressive symptoms are closely intertwined and constitute two key dimensions of mental health in late childhood and early adolescence. Although robust associations between these constructs are well documented, less is known about how they influence one another over time at the level of specific symptoms. Examining their longitudinal dynamics can clarify whether—and which—elements of loneliness precede increases in particular depressive symptoms (and vice versa), thereby moving beyond global score correlations. Such symptom-level insight is essential for identifying actionable targets for timely, developmentally sensitive preventive interventions.

### 1.1. Depression

Depression is a prevalent mental disorder in the general population (COVID-19 Mental Disorders Collaborators, 2021) and notably among children and adolescents (Merikangas et al., 2009). It can manifest in children as young as 3 (Mullen, 2018), though it is rare in preschoolers, with a 1.1% prevalence rate (Vasileva et al., 2021). However, the frequency of depression significantly increases in the teenage years, especially after age 11 (Lewinsohn et al., 1993), when it reaches a rate of 2.6% (Polanczyk et al., 2015), with about 20% of young people experiencing it by age 19 (Lu, 2019). Note that approximately two-thirds of lifetime depression cases among adults emerge in the teenage years (Kessler et al., 2005), beginning typically between ages 11 and 14, making this phase crucial to understanding the origin of this disorder (Gijzen et al., 2021).

Childhood depression is linked to many long-term negative outcomes. It affects academic performance and reduces opportunities for higher education and employment. It is also associated with negative emotions, low self-esteem, suicidal tendencies, substance abuse, and physical health issues, leading to financial costs (Grygiel et al., 2023). Youth depression may stem from various causes (Abela & Hankin, 2008), and—in line with the interpersonal theory of depression (Coyne, 1976)—one of its more important sources may be problems in interpersonal relationships, expressed in loneliness (Rudolph, 2008).

### 1.2. Loneliness

Loneliness has been described as “a sad or aching sense of isolation” (Parkhurst & Hopmeyer-Gorman, 1999, p. 58), and is characterized as a distressing emotional state arising from unmet intimacy needs and a disparity between expected and actual social relationships (Asher & Weeks, 2013). However, loneliness is not a unitary experience. The literature commonly distinguishes between emotional loneliness, which refers to the perceived absence of close, intimate, or attachment-related relationships, and social loneliness, which reflects a perceived lack of belonging to a broader social network, peer group, or community (Maes et al., 2022; Weiss, 1973). Other accounts also emphasize existential loneliness, understood as a deeper sense of separateness, disconnection, or not being fully understood by others (Mansfield et al., 2021).

These distinctions are relevant for research on depression because different forms of loneliness may not be equally related to depressive symptoms. Loneliness involving emotional pain, rejection, lack of close relational bonds, or a sense of being unwanted may be especially closely connected with depressive affect, such as sadness, worthlessness, or hopelessness. By contrast, broader social loneliness may be linked to depression through partly different interpersonal pathways, such as low social integration, limited peer belonging, or reduced perceived social support. Thus, the relationship between loneliness and depression may depend not only on the overall level of loneliness, but also on the specific aspect of loneliness being considered.

Loneliness also differs in how it is measured. Direct measures explicitly ask respondents whether or how often they feel lonely, whereas indirect measures assess experiences associated with loneliness, such as lack of closeness, perceived isolation, or feeling left out, without using the term “lonely” itself (Shiovitz-Ezra & Ayalon, 2012). Direct and indirect measures are related, but they are not fully interchangeable and may capture partly different aspects of the construct. This distinction is important because direct measures may be closer to explicitly recognized feelings of loneliness, whereas indirect measures may better reflect broader evaluations of one’s relational situation (Barreto et al., 2022).

Research indicates that loneliness peaks among teenagers and older adults, with about 10% of school-age children experiencing it (Mund et al., 2020). Like depression, loneliness has numerous adverse effects on teenagers. It affects school engagement, motivation, and outcomes, reduces well-being, and is linked to aggression, suicidal tendencies, substance use, problems with sleeping, various physical health risks, and a broad spectrum of mental disorders such as anxiety (Grygiel et al., 2019; Martín-Rodríguez et al., 2022).

### 1.3. The Interplay Between Loneliness and Depression

Since the 1970s, studies have linked loneliness to depression (Russell et al., 1978). Recent meta-analyses reveal a moderate correlation between them in both the general population (*r* = 0.50; Erzen & Çikrikci, 2018; *r* = 0.49; Park et al., 2020); and children/adolescents (*r* = 0.48; Dunn & Sicouri, 2022; *r* = 0.61; Mahon et al., 2006). While these two experiences might share common causes and features (Heinrich & Gullone, 2006), factor analyses indicate that they are distinct constructs (Cacioppo et al., 2006).

Unfortunately, there is still a lack of clarity about which symptoms of depression are most strongly associated with loneliness. This lack of clarity can be attributed in part to the dominant perspective in the understanding of psychopathological disorders, which is the latent perspective. A latent approach implicitly assumes that mental disorders such as depression cause feelings of sadness or worthlessness in the same way that the smallpox virus causes pustules, fever, and headache (Beard et al., 2016). From a latent modeling perspective, the causes and consequences related to the underlying latent variable itself are more important than specific symptoms. In practice, from a latent perspective, constructs (such as depression) are aggregate measures (most often formed as the sums/means of the items that form a given scale).

In contrast, the psychometric network perspective sees mental disorders as systems in which symptoms interact and often amplify each other (Borsboom & Cramer, 2013). Symptoms are not merely outcomes of a latent cause but play an active role; they influence and trigger each other rather than stemming from a singular origin (Fried, 2015). This viewpoint suggests that mental disorders emerge from intricate symptom interactions rather than a singular underlying (latent) cause (Fried & Nesse, 2014). Recognizing the varying importance of individual symptoms can provide insight into their clinical relevance and provide a more nuanced understanding of the etiology of mental disorders (Hofmann & Curtiss, 2018).

### 1.4. Results and Limitations of Previous Research on Depressive Symptoms Networks and Loneliness Among Teenagers

Considering the significance of depression and loneliness in the development of teenagers, it is notable how infrequently the network relationships between them have been studied. Only sixteen studies from thirteen articles have addressed this topic (see Table S1 in Supplementary Materials).

These studies consistently highlight loneliness as a key component of the depressive symptoms network, often ranking it as the first or second most central element. This pattern underscores the robust and theoretically relevant association between loneliness and adolescent depression, while remaining consistent with meta-analytic evidence indicating a moderate association between these constructs. Moreover, they show that loneliness is closely tied to crying, feelings of sadness, worthlessness, unhappiness, and suicidal ideation. As a result, they provide evidence that loneliness is linked to depression, primarily via a negative cognitive and emotional style that triggers a sad or depressed mood (Ciarrochi & Heaven, 2008), a core symptom of depression (Hoffart et al., 2021). This insight greatly improves our understanding of the relationship between loneliness and depression.

At the same time, all these network analyses have been cross-sectional, depicting associations among symptoms at a single point in time, which precludes the making of claims (Ployhart & Vandenberg, 2010) about the direction of the relationships. Thus, there are no solid empirical grounds for identifying whether an increase in loneliness leads to more sadness, or whether sadness contributes to an increase in loneliness. Consequently, although cross-sectional research can identify loneliness as a significant element in the network of symptoms of depression and its associations with specific symptoms at a given time, it does not offer a solid foundation for hypothesizing causal relationships.

Since cross-sectional data represent only one moment in time, they do not meet the “causality” assumption that causes must precede effects (Lewis, 1973). Given this, cross-sectional analysis relies on theoretical inferences to make arguments about causality (Kearney, 2017). A partial remedy to such threats to internal validity can be provided by employing longitudinal designs and data analysis with methods such as cross-lagged panel modelling (CLPM; Finkel, 1995; Kenny, 1975), which is often considered to be the most appropriate approach to the study causality when compared to other longitudinal models in non-experimental data (Hamaker et al., 2015).

Although traditional cross-lagged panel models (CLPMs) are useful for examining prospective associations between variables, they are less well suited to the analysis of symptom networks because they do not naturally represent multiple symptom-to-symptom relations as a directed network. The cross-lagged panel network model (CLPN) addresses this limitation by combining network methodology with the logic of cross-lagged panel modeling (Wysocki et al., 2022). CLPN estimates directed paths from one measurement occasion to the next while adjusting for previous levels of all variables in the network. It therefore allows researchers to examine both autoregressive effects, reflecting rank-order stability in a given symptom, and cross-lagged effects, indicating whether individual differences in one symptom predict subsequent rank-order changes in another symptom. In contrast to undirected cross-sectional networks, CLPN provides a longitudinal network of directed prospective associations. However, as with traditional CLPMs, these paths should be interpreted as prospective associations rather than definitive evidence of causality (Kearney, 2017; Wysocki et al., 2022).

### 1.5. Current Study

The present study used a cross-lagged panel network approach to examine the longitudinal interplay between loneliness and depression-related affective and behavioral states in late childhood. Rather than treating depressive symptoms as interchangeable indicators of a single latent construct, we focused on prospective symptom-level associations between loneliness and specific emotional and behavioral experiences assessed in the NLSY79 Child Self-Administered Supplement, including sadness, nervousness/tension, happiness, boredom, fatigue, busyness, and perceived parental pressure. This approach allowed us to examine whether loneliness predicts later changes in specific depression-related states, whether these states predict later loneliness, and whether some associations are reciprocal.

To address the lack of longitudinal symptom-level evidence, we derived several theory-driven hypotheses concerning selected prospective associations. First, because loneliness reflects perceived interpersonal disconnection and unmet belongingness needs (Asher & Weeks, 2013; Baumeister & Leary, 1995), and because sadness may both result from and further impair social relationships (Leary, 2015), we expected a reciprocal positive longitudinal association between loneliness and sadness (H1). This expectation was also consistent with previous cross-sectional network studies showing strong associations between loneliness and sadness-related symptoms in youth depression networks (Gijzen et al., 2021; Kim et al., 2021; Mullarkey et al., 2019).

Second, because positive affective states may support social engagement, prosocial orientation, and better interpersonal functioning (Diener & Seligman, 2002; Myers, 2000; Saldarriaga et al., 2015), we expected higher happiness at baseline to predict lower loneliness at follow-up (H2). However, because positive and negative affect are not simply opposite poles of one dimension, but may operate as partly independent affective systems, we did not necessarily expect loneliness to predict a subsequent decline in happiness (Cacioppo et al., 1999; Ong et al., 2017; Rafaeli & Revelle, 2006).

Third, because loneliness is associated with heightened social vigilance, threat sensitivity, and emotional distress (Lieberz et al., 2021; Park et al., 2020; Vanhalst et al., 2018), we expected loneliness to predict later nervousness, tension, or edginess (H3). Fourth, because boredom reflects disengagement from meaningful activity, emotional discomfort, and difficulty sustaining satisfying involvement with the environment (Eastwood et al., 2012; Goldberg et al., 2011), and because boredom may reduce the perceived value of social interactions (Sommers & Vodanovich, 2000; Vodanovich, 2003), we expected boredom to predict higher subsequent loneliness (H4).

Fifth, because perceived parental pressure represents a salient interpersonal stressor in late childhood and adolescence (Kocayörük et al., 2015; Ruhl et al., 2015), and because lonely children may be more likely to interpret parental involvement as controlling or pressuring (Lin et al., 2020), we expected reciprocal positive longitudinal associations between parental pressure and loneliness (H5). Given the scarcity of longitudinal symptom-level network studies on loneliness and depressive symptoms in children, the remaining cross-lagged associations were treated as exploratory. Thus, the study combined theory-driven hypotheses concerning selected directional paths with an exploratory network approach aimed at identifying additional prospective symptom-level connections.

## 2. Materials and Methods

### 2.1. Data Source and Sample

We analyzed data from the National Longitudinal Surveys of Youth 1979 Children and Young Adults (NLSY79-CY), administered by the U.S. Department of Labor. The original NLSY79 began in 1979 and included approximately 6300 young women aged 14–21 years. Beginning in 1986, the biological children of female NLSY79 respondents were assessed biennially. Children aged 10–14 completed the Child Self-Administered Supplement (CSAS). Since 1996, the CSAS has included depression-related items derived from the National Commission on Children 1990 Survey of Parents and Children (National Commission on Children, 1999). In contrast, adolescents aged 15 years and older completed a different questionnaire that included a different depression scale, the 7-item CES-D.

Because the present study focused on late childhood and required comparable symptom-level indicators across two consecutive measurement occasions, we restricted the analysis to children aged 10–12 years at baseline. This age restriction ensured that children completed the same depression-related questionnaire at both baseline and follow-up. Children who first completed the CSAS at ages 13–14 would be 15–16 years old at the next biennial assessment and would therefore complete the older-adolescent questionnaire, making the depression-related items non-comparable across time.

**Figure 1:**
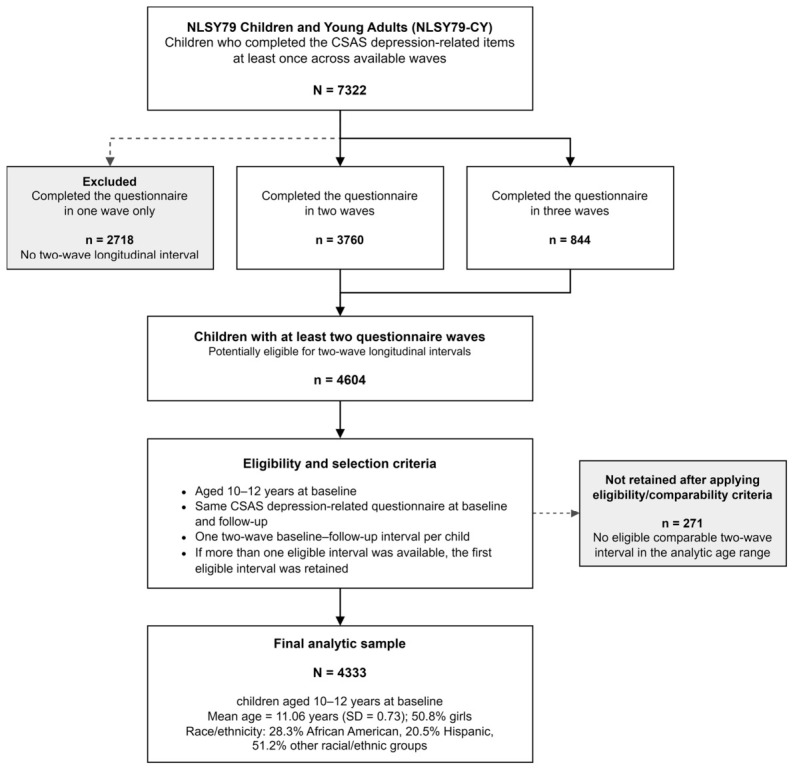
Flowchart of participant selection for the analytic sample. Note. NLSY79-CY = National Longitudinal Surveys of Youth 1979 Children and Young Adults; CSAS = Child Self-Administered Supplement.

As summarized in Figure 1, participant selection proceeded in several steps. First, all children who completed the relevant CSAS depression-related items in at least one wave were identified. Across the available waves, 7322 children completed the questionnaire at least once. Of these, 2718 participated in one wave only, 3760 participated in two waves, and 844 participated in three waves. Second, we identified all eligible two-wave intervals in which the same depression-related questionnaire was completed at two consecutive measurement occasions. Third, because some children, particularly those aged 10 or 11 years at their first eligible assessment, could contribute more than one eligible two-wave interval, we retained only the first eligible two-wave interval for each child. Thus, each child included in the analytic sample contributed two consecutive observations—baseline and follow-up—but was represented only once in the longitudinal analyses.

More specifically, all children who participated in the first eligible two-wave interval (1992→1994) were included. For the next interval (1994→1996), children who had already been included from the previous interval were excluded. The same rule was then applied sequentially to each subsequent pair of consecutive waves. Thus, the final analytic sample included all available children who met the age and questionnaire-comparability criteria and contributed one unique two-wave observation interval, consisting of a baseline and a follow-up assessment.

The final sample consisted of 4333 children aged 10–12 years at baseline (*M*_age_ = 11.06, *SD* = 0.73; 50.8% girls). The sample included 28.3% African American children, 20.5% Hispanic children, and 51.2% children from other racial/ethnic groups.

### 2.2. Measures

Children completed one item assessing loneliness and eight depression-related affective and behavioral items from the Child Self-Administered Supplement of the NLSY79-CY. The items were derived from the National Commission on Children’s 1990 Survey of Parents and Children (National Commission on Children, 1999). Children were asked how often they felt: (a) lonely, (b) sad and blue, (c) nervous, tense, or on edge, (d) happy, (e) bored, (f) tired or worn out, (g) excited about something they were looking forward to, (h) too busy to get everything done, and (i) pressured by their mother or father. Responses were given on a 3-point scale ranging from hardly ever to often (see Table S2 in Supplementary Materials).

For descriptive psychometric purposes, we estimated the internal consistency of the eight depression-related items, excluding the loneliness item, at both measurement occasions. Because the items were ordinal, internal consistency was assessed using ordinal alpha based on the polychoric correlation matrix. The positively worded items assessing happiness and excitement were reverse-coded for this reliability analysis so that higher scores consistently reflected greater depression-related difficulties. The eight-item set showed modest internal consistency, with ordinal alpha coefficients of α = 0.65 at T1 and α = 0.69 at T2. These coefficients should be interpreted as descriptive psychometric information only, because the main CLPN analyses treated the items as separate symptom-level indicators rather than as interchangeable indicators of a single latent scale. Because loneliness was assessed with a single item, internal consistency could not be estimated for this variable. The distribution of the loneliness item at both measurement occasions is reported in Table S2, and the limitations of single-item loneliness assessment are discussed in Section 4.8.

### 2.3. Statistical Analyses

#### 2.3.1. Item Redundancy Analysis

The main analyses were preceded by preliminary ones in order to identify redundant items. A key assumption of network psychometrics is that network elements (nodes) are unique causal components, meaning that nodes are not exchangeable with other nodes of the network (Christensen et al., 2020). As a result, these elements should be clearly distinct, rather than redundant. Overlapping items can misrepresent relationships between nodes (symptoms) and affect the bias of various network measures, e.g., centrality (Hallquist et al., 2021).

Redundant pairs of items were evaluated using Unique Variable Analysis (UVA) (Christensen et al., 2023). This procedure identifies local dependence using network modeling and a graph theory measure called weighted topological overlap (wTO). wTO quantifies the ‘overlap’ of items in a network by computing the similarity of their connections (number, strength, and sign). The redundant pairs of items were identified as those in which the wTO exceeded—as suggested in simulation studies—the cutoff value of 0.25 (Christensen et al., 2023). This value was used as a simulation-based screening threshold proposed by Christensen et al. (2023) for detecting potentially redundant item pairs. To make the redundancy decision more conservative, we treated an item pair as redundant only when it exceeded this threshold at both measurement occasions and when the overlap was also substantively meaningful. The UVA approaches were implemented using the EGAnet package in R, version 1.1.0 (Golino et al., 2022).

#### 2.3.2. Longitudinal Network (Cross-Lagged Panel Network—CLPN)

In the main part of the analysis, the relationships between loneliness and depressive feelings over time were examined using a cross-lagged panel network (CLPN; Wysocki et al., 2022). A CLPN’s primary characteristic is that it models relationships between items over time as directed paths. These paths represent the shared variance between a variable at one time point and another (or the same) variable at the next, factoring in all other variables at the initial time point. Let us emphasize that, contrary to undirected networks in cross-sectional data, CLPN paths move from one measurement occasion to a subsequent one (Wysocki et al., 2022).

To minimize the chances of false edges in the network models, least absolute shrinkage and selection operator (LASSO) regularization was used. This adjustment to regression coefficients helps prevent overfitting and simplifies the network structure. Therefore, this approach decreases the quantity of non-zero regression coefficients (edges). The LASSO regularized regression and 10-fold cross-validation were conducted using the glmnet package in R (Friedman et al., 2010). The analyses used lambda penalization value (lambda$min), thereby minimizing the mean cross-validated error.

In CLPN the edge weights can be interpreted similarly to a regression coefficient in the traditional cross-lagged model (CLPM). As in CLPM, in CLPN autoregressive (also referred to as stability effect, inertia, tendency to not move) and cross-lagged effects are estimated.

The autoregressive effect refers to the coefficient in which a node at a given time point predicts its own value at the next time point, taking into account all other nodes at that time. In other words, information is provided on the rank-order stability of the constructs (i.e., stability of interindividual differences) from one occasion to the next.

Cross-lagged effects indicate whether and how strongly nodes in an initial assessment (T1) predict the values of other nodes at the subsequent stage of assessment (T2), while accounting for all other variables at the starting assessment (T1) and autoregressive effects for the outcome variable. Finding a non-zero cross-lagged coefficient may indicate predictive relations over time and suggest, though not prove, the presence of causal relationships (Wysocki et al., 2022). For example, a positively cross-lagged coefficient between two constructs (loneliness and sadness) will imply that, when respondents have a high position on the loneliness scale (relative to other respondents), they will experience a subsequent rank-order increase in sadness compared to individuals with high loneliness. Therefore, in CLPN depression symptoms and their correlates can be represented as dynamic with interactive indicators, such that, for instance, previously low self-esteem leads to a later increase in loneliness, and previous loneliness fosters a later higher intensity of sadness.

In addition to autoregression coefficients and cross-lagged effects, strength centrality was estimated, which is the sum of the absolute values of the cross-lagged effects. This consists of in-strength (in-prediction) and out-strength (out-prediction). The in-strength indicates the relative prospective predictability of the target node and is calculated as the sum of the absolute values of incoming (cross-lagged) edges associated with one symptom. The out-strength highlights its relative influence over time and is calculated as the sum of the absolute values of outgoing edges associated with one symptom. Note that the autoregressive path is omitted when computing outgoing and incoming strengths.

## 3. Results

Before estimating the temporal network, we examined descriptive changes in the study variables and their bivariate associations, followed by an item redundancy analysis to determine the final set of network nodes.

### 3.1. Descriptive Statistics and Correlations

The severity of the symptoms between T1 and T2 (over two years) remained stable (Table S2 in Supplementary Materials). Small, statistically significant differences (Cohen’s *d* < 0.07) were observed for ‘sad’, ‘bored’, ‘lonely’, and ‘pressured’. The intensity decreased for ‘sad’, ‘lonely’, and ‘pressured’, but increased for ‘bored’.

Spearman correlations for the instruments administered to the study participants are presented in Table S3 in Supplementary Materials. These coefficients range from −0.28 to 0.39. The average of the absolute values of the correlation coefficients was 0.13 (*SD* = 0.08). Out of the 153 coefficients, only 13 were not statistically significant (*p* > 0.05).

### 3.2. Redundancy Analysis

Initially, we examined redundancies between item pairs based on the similarity of their connections with other nodes using Unique Variable Analysis (UVA). Consistent with the decision rule described above, an item pair was considered redundant only if its weighted topological overlap (wTO) exceeded the simulation-based threshold of 0.25 at both measurement occasions and if the overlap was substantively meaningful. Using this rule, the only item pair that met the redundancy criteria was “happy” and “excited about something they were looking forward to” (wTO = 0.32 at T1; wTO = 0.41 at T2). Because both items reflected closely related positive affective states, they were averaged into a composite node labelled “happiness” in the subsequent CLPN analyses. As a result, the final network consisted of eight nodes.

### 3.3. Temporal (Cross-Lagged Panel) Network

**Figure 2:**
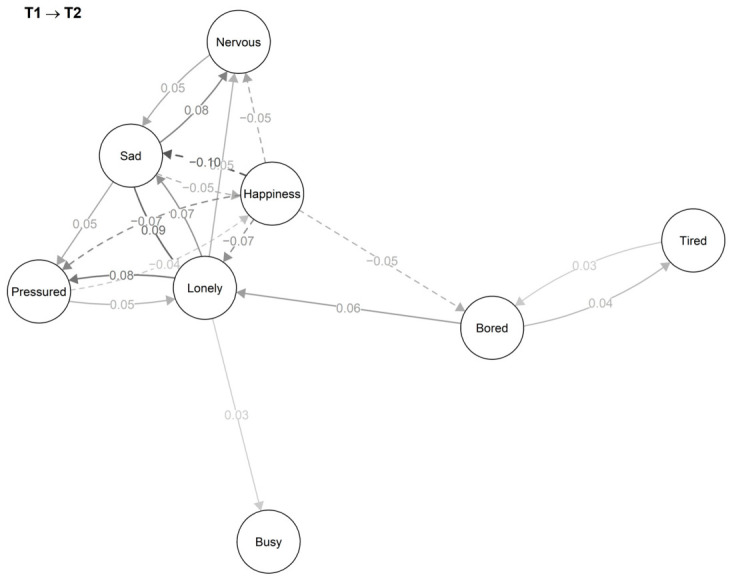
Longitudinal cross-lagged panel network of symptoms between T1 and T2. Note. Each node represents the same construct measured at both waves. Arrows represent cross-lagged paths from a variable at T1 to a different variable at T2, after adjusting for all other T1 variables and the autoregressive effect of the outcome variable. For example, an arrow from lonely to sad indicates that loneliness at T1 predicts sadness at T2. Autoregressive paths are omitted to improve readability. Edge labels are standardized cross-lagged regression coefficients, not correlations or covariances. Solid lines indicate positive coefficients, whereas dashed lines indicate negative coefficients. The thickness of an edge reflects the absolute magnitude of the coefficient. Filled points indicate statistically significant cross-lagged paths at *p* < 0.05; open points indicate non-significant paths.

The estimated cross-lagged panel network is presented in Figure 2, with autoregressive edges omitted to improve readability. The network shows directed prospective associations from T1 to T2 after adjusting for all baseline nodes and the autoregressive effect of each outcome node. Overall, the network indicated several theoretically meaningful longitudinal links between loneliness and depression-related affective and behavioral states.

**Figure 3:**
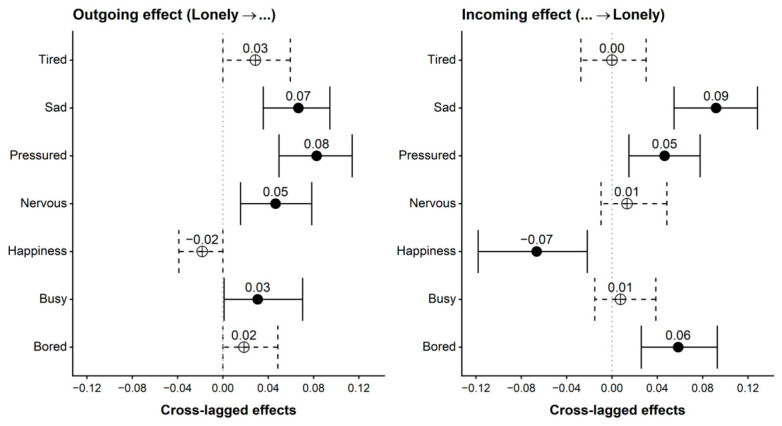
In-prediction and out-prediction of the loneliness in cross-lagged network (T1→T2). Note. The left panel presents outgoing effects from loneliness at T1 to other variables at T2. The right panel presents incoming effects from other variables at T1 to loneliness at T2. Points represent standardized cross-lagged regression coefficients. The numbers next to the points are the estimated coefficients. Horizontal lines represent 95% confidence intervals around the estimates. The vertical dashed line indicates zero, that is, no cross-lagged effect. Filled black points with solid horizontal lines indicate statistically significant effects at *p* < 0.05, whereas open points with dotted horizontal lines indicate non-significant effects. Positive coefficients indicate that higher levels of the predictor at T1 are associated with higher levels of the outcome at T2, after controlling for the remaining T1 variables and the autoregressive effect of the outcome. Negative coefficients indicate that higher levels of the predictor at T1 are associated with lower levels of the outcome at T2.

The most relevant cross-lagged paths involving loneliness are summarized in Figure 3. Loneliness showed significant reciprocal associations with sadness and perceived parental pressure. Specifically, higher loneliness at T1 predicted higher sadness and higher perceived parental pressure at T2, whereas both sadness and perceived parental pressure at T1 predicted higher loneliness at T2. In addition, loneliness at T1 predicted higher nervousness/tension and busyness at T2. Conversely, higher boredom at T1 predicted higher loneliness at T2, whereas higher happiness at T1 predicted lower loneliness at T2. Loneliness did not significantly predict later boredom or happiness.

Stability and centrality analyses supported the robustness and interpretability of the estimated network. Edge stability was high, CS (cor = 0.70) = 0.75, indicating stable estimation of the network structure. Out-strength also showed acceptable stability, CS (cor = 0.70) = 0.52, whereas in-strength was less stable, CS (cor = 0.70) = 0.21; therefore, in-strength results should be interpreted more cautiously. These stability results are reported in Supplementary Figures S1 and S2.

**Figure 4:**
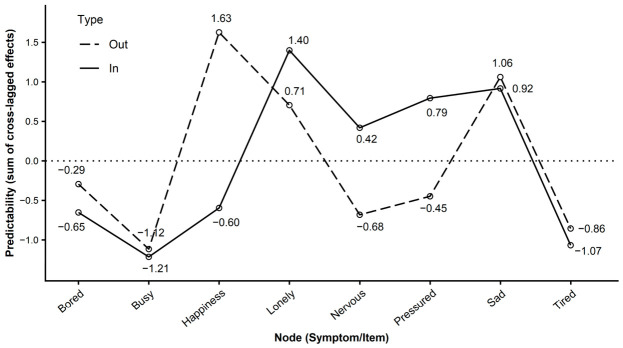
Cross-lagged in-prediction and out-prediction centrality of variables in the estimated CLPN (T1→T2). Note. Out indicates how strongly a node predicts other nodes in the network; In indicates how strongly a node is predicted by other nodes.

Autoregressive effects, presented in Supplementary Figure S3, were generally modest, with a mean value of 0.16. The highest autoregressive effect was observed for boredom (0.23), whereas the lowest was observed for fatigue (0.10). Loneliness showed slightly above-average stability (0.18). Centrality results, presented in Figure 4, indicated that happiness, sadness, and loneliness had relatively high outgoing influence, whereas loneliness showed the highest incoming predictability. Thus, loneliness functioned both as a predictor of later depression-related states and as an outcome predicted by earlier emotional and interpersonal experiences.

Bootstrapped difference tests indicated that the cross-lagged edge weights did not significantly differ from one another in strength, and the accuracy of edge estimates is shown in Supplementary Figures S4 and S5.

## 4. Discussion

The purpose of our study was to examine the reciprocal associations between loneliness and depression-related affective and behavioral states in late childhood using a cross-lagged panel network model. Overall, the findings supported the hypothesized reciprocal association between loneliness and sadness (H1), the negative prospective association between happiness and later loneliness (H2), the positive prospective association between loneliness and later nervousness/tension (H3), the positive prospective association between boredom and later loneliness (H4), and the hypothesized reciprocal associations between perceived parental pressure and loneliness (H5). In addition, the model identified an exploratory prospective path from loneliness to later busyness, suggesting that lonely children may become more likely to experience themselves as too busy to get everything done over time.

These findings shed light on the complex interplay between loneliness and several depression-related affective and behavioral states, including negative affective states such as sadness and nervousness/tension, positive affective states such as happiness, activity- and fatigue-related experiences such as boredom, fatigue, and busyness, and perceived interpersonal pressure in the family context. Importantly, loneliness emerged both as a predictor of later depression-related states and as an outcome predicted by earlier emotional and interpersonal experiences.

### 4.1. Sadness

Our study revealed a bidirectional association between loneliness and sadness, a core symptom of depressive mood. This finding supported H1 and suggests that these two affective experiences may reinforce each other over time. Sadness is a complex natural emotional state characterized by feelings of unhappiness, often arising from everyday setbacks and disappointments or from a sense of loss related to relationship problems and difficulties in social interaction (Bondolfi et al., 2015; Leary, 2015). From this perspective, loneliness may increase later sadness by making interpersonal disconnection and unmet belongingness needs more salient.

The reverse prospective effect, from sadness to later loneliness, is also theoretically plausible. First, sadness may affect loneliness through its negative impact on peer status and social functioning. Emotional expression has a communicative function (Scarantino et al., 2022), affects beliefs about the expresser (Tiedens, 2001), and can be an important predictor of peer status (Denham et al., 2001). Expressions of negative affect are negatively associated with social competence and peer group status (Walter & LaFreniere, 2000). Sad individuals may be perceived as more passive, behaviorally inhibited (Frijda, 1986), and less socially competent (Jones et al., 2002), which may reduce their attractiveness as interaction partners and increase later loneliness.

Second, sadness may increase loneliness by affecting how children interpret and experience their relationships, irrespective of the objective state of those relationships. People experiencing higher levels of sadness may generate more negatively biased ratings of their social functioning compared with observer ratings (Durand et al., 2021). Moreover, because sadness can also be triggered by non-social factors, such as health-related losses or other adverse experiences (Atherton et al., 2015), it may cast a broader negative tone over the perception of interpersonal relationships. This interpretation is consistent with Beck’s cognitive theory, which suggests that negative self-perception can shape one’s view of relationships (Beck, 1979). Thus, sadness and loneliness may become linked through both interpersonal behavior and biased relational appraisal, although further research is needed to test these mechanisms directly.

### 4.2. Happiness

Our analysis also found a unidirectional prospective association from happiness to lower subsequent loneliness, supporting H2. This finding suggests that positive affective states may play a protective role in relation to later loneliness. Prior research indicates that happier individuals tend to be more extraverted, agreeable, and less prone to neuroticism (Diener & Seligman, 2002). They are also generally described as less self-focused and more compassionate, forgiving, trusting, energetic, decisive, creative, sociable, and helpful (Myers, 2000). These qualities may support stronger and more intimate interpersonal connections (Saldarriaga et al., 2015), thereby reducing later loneliness.

Interestingly, loneliness did not predict a subsequent decline in happiness, in contrast to the dynamics observed for sadness. This finding is important because loneliness is associated with an unmet need for belonging, which is relevant to happiness (Baumeister & Leary, 1995; Saldarriaga et al., 2015). At the same time, the absence of a reverse path is consistent with theories of emotional complexity, according to which positive and negative emotions are not merely opposite ends of a single continuum but represent partly distinct emotional dimensions (Ong et al., 2017; Rafaeli & Revelle, 2006; Ready et al., 2008). The notion of partially independent affective systems is also illustrated by the uncoupled activation hypothesis, according to which positive and negative affect can be activated independently rather than necessarily changing in opposite directions (Cacioppo et al., 1999). Thus, loneliness may be more closely linked to later negative affective states, such as sadness or nervousness, than to a later reduction in positive affective states such as happiness.

### 4.3. Irritable Mood (“Nervous, Tense, or on Edge”)

Our study also identified a unidirectional effect of loneliness on irritable mood manifesting as feelings of nervousness, tension, or edginess (Stringaris et al., 2013). Loneliness, as an unpleasant (distressing) emotional condition characterized not only by sadness but also by anxiety (Park et al., 2020; Vanhalst et al., 2018), often results in heightened social vigilance and distrust in social situations (Lieberz et al., 2021), leading to cognitive distortions in interpreting social cues as threats. Then, such distortions can contribute to irritability, which is also recognized as a symptom of depressive mood, as well as anxiety disorders causing social withdrawal (Stringaris, 2011).

Simultaneously, we did not find evidence that irritability leads to increased loneliness. This suggests that the vicious cycle between loneliness and irritability, in which irritability may reduce the likelihood of forming satisfying relationships and increase feelings of isolation, may require more time to develop. Therefore, future research on this relationship should consider longer time frames, as demonstrated by Danneel et al. (2019).

### 4.4. Keeping Busy

Our findings showed an exploratory unidirectional prospective association from loneliness to later busyness. This path was not specified a priori and should therefore be interpreted cautiously. The item “too busy to get everything done” does not indicate the type, context, or intentional function of the activities involved. Consequently, the present data do not allow us to determine whether later busyness reflects adaptive engagement, external demands, avoidance, distraction, or an attempt to cope with loneliness.

One possible interpretation is that children who feel lonely may later experience greater subjective overload or become more involved in activities that make them feel busy. Prior research suggests that lonely children and adolescents may spend solitary time in different ways, including passive screen time, gaming, watching TV, music, reading, homework, writing, drawing, cycling, or running (Coplan et al., 2021; Li et al., 2017). Such activities may provide a sense of engagement or temporary relief from distress, but in the present study this mechanism was not directly tested. Moreover, although solitary activities are not necessarily maladaptive and may sometimes be experienced as fulfilling or emotionally regulating, their function depends on their content, motivation, and context (Coplan et al., 2015; Verity et al., 2022). Therefore, busyness should not be interpreted as a confirmed coping mechanism. Future studies should examine whether the activities that follow loneliness are social or solitary, voluntary or externally imposed, and whether they reduce, maintain, or intensify loneliness over time.

### 4.5. Boredom

Our analysis revealed a significant unidirectional link between boredom and increased feelings of loneliness. Previous research suggests that boredom is not merely a result of inactivity; rather, it reflects a deeper psychological discomfort, encompassing sadness, emptiness, anxiety, lack of motivation, and emotional disengagement (Eastwood et al., 2012; Goldberg et al., 2011). It stems from an inability to engage in fulfilling activities, leading to frustration and dissatisfaction. Although boredom can initially motivate people to seek rewarding experiences (Van Tilburg & Igou, 2017), chronic boredom may signal difficulties in adapting to one’s environment and engaging effectively in daily life (Todman, 2003).

Individuals prone to boredom often show lower self-control and may engage in negative or unproductive behaviors. This can result in feelings of disconnection from their surroundings and a reduced sense of control over their lives (Eastwood et al., 2012; Goldberg et al., 2011). Such feelings may increase loneliness because social interactions may be perceived as less satisfying, less meaningful, or even trivial (Vodanovich, 2003), potentially contributing to less fulfilling relationships and feelings of rejection (Ayhan et al., 2021; Sommers & Vodanovich, 2000).

Interestingly, loneliness did not predict later boredom. This suggests that the boredom–loneliness association observed in the present model was directional rather than reciprocal over the two-year interval. One possible explanation is that boredom reflects a broader difficulty in engaging with the environment, including the social environment, whereas loneliness may be followed by different forms of activity, withdrawal, or subjective overload that are not captured by the boredom item. However, because the present model did not test mediating mechanisms and did not assess the content of children’s activities, this interpretation remains tentative. Future research should examine whether boredom contributes to loneliness through reduced social engagement, lower perceived meaning of social interactions, or withdrawal from rewarding activities.

### 4.6. Parental Pressure

Finally, our analysis identified a bidirectional association between perceived parental pressure and loneliness. This was one of the clearest reciprocal patterns in the network and is consistent with the possibility of a mutually reinforcing interpersonal process in the family context. On the one hand, perceived parental pressure may represent a salient interpersonal stressor. Excessive parental expectations regarding academics, extracurricular activities, or social behavior can make adolescents feel misunderstood and may contribute to withdrawal from family interactions and lowered self-esteem (Kocayörük et al., 2015; Ruhl et al., 2015). Adolescents with diminished self-worth may, in turn, struggle with feelings of rejection and isolation not only within their families but also in peer interactions (Pinquart, 2022). This may contribute to later loneliness.

On the other hand, loneliness may also increase later perceptions of parental pressure. One possible explanation is that lonely adolescents may become more sensitive to parental guidance and interpret it as excessive pressure. Parents may also respond to a child’s withdrawal with greater involvement or control, which the adolescent may experience as additional pressure (Lin et al., 2020). This interpretation is consistent with the idea of a reciprocal family process, but it should remain cautious because the item assessed children’s perceived pressure from their mother or father, not parental behavior directly. Thus, the observed association may reflect actual parental demands, children’s subjective interpretation of parental involvement, or both. Future studies should include more differentiated measures of family climate, parental control, emotional support, self-esteem, and children’s perceptions of parental expectations.

### 4.7. Practical Implications

Our analyses confirm relationships between loneliness and depressive symptoms. These dependencies are crucial for understanding the effectiveness of therapeutic interventions. To effectively support the mental health of children and adolescents, a holistic approach is needed—one that focuses not only on reducing negative emotions but also on promoting positive experiences and enhancing social and emotional skills.

Cognitive-behavioral therapy (CBT) can simultaneously address sadness and loneliness by improving social skills and coping strategies, thus reducing sadness associated with loneliness (Masi et al., 2011). Well-being programs utilizing mindfulness-based stress reduction (MBSR) and positive psychology techniques can foster happiness and resilience, effectively reducing loneliness (Teoh et al., 2021). Group therapies, such as Social Skills Training (SST; De Mooij et al., 2020), Dialectical Behavior Therapy (DBT; Pardo et al., 2020), or Interpersonal Therapy (IPT; Lipsitz & Markowitz, 2013), provide a supportive environment for improving social interactions, which can alleviate anxiety related to loneliness.

Parenting workshops, such as the P-Positive Parenting Program, which emphasize effective communication and empathetic parenting, can help balance parental expectations with support, reducing children’s feelings of pressure and consequently their loneliness (Sanders et al., 2000). These workshops, by promoting compassion and understanding, contribute to creating a more supportive home environment.

Another effective strategy is engaging children and adolescents in organized activities such as hobby clubs, community projects, sports, or volunteering. These activities not only combat chronic boredom but also foster meaningful social connections, providing opportunities for valuable interactions and relationships. Research shows that structured use of leisure time, such as learning a new hobby, effectively reduces feelings of loneliness in children and adolescents (Eccles & Qualter, 2021).

### 4.8. Limitations

This study on the longitudinal interplay between loneliness and depressive feelings in children and adolescents has some limitations. First, the study is constrained by a correlational research model (non-experimental design) based on self-reported data at only two time points, limiting our ability to establish causality and fully capture the dynamics of the observed relationships.

Second, loneliness was assessed with a single direct self-report item: “How often do you feel lonely?” This is an important limitation. Direct measures explicitly ask respondents whether or how often they feel lonely, whereas indirect measures assess experiences associated with loneliness, such as lack of closeness, perceived isolation, or feeling left out, without using the term “lonely” itself (Shiovitz-Ezra & Ayalon, 2012). Although direct single-item measures have high face validity and are practical in large-scale longitudinal surveys, they cannot capture the multidimensional nature of loneliness and may be vulnerable to under-reporting because loneliness can be stigmatized (Barreto et al., 2022). Moreover, direct and indirect measures of loneliness are related but not fully interchangeable; they may classify different respondents as lonely and capture partly different aspects of the construct (Eccles et al., 2020; Shiovitz-Ezra & Ayalon, 2012). Thus, the item used in this study may primarily reflect children’s explicit recognition of feeling lonely. The use of alternative measures, such as validated multi-item scales (e.g., the UCLA Loneliness Scale or the De Jong Gierveld Loneliness Scale; Maes et al., 2022), could provide a more nuanced understanding of this complex emotion.

Third, our study is based on a non-standard measure of depressive symptoms. Subsequent studies should use measures consistent with the DSM classification, such as the General Health Questionnaire-9 modified for adolescents (Johnson et al., 2002). Furthermore, a notable limitation of this study is its focus on children aged 10–12 years. Given the significant psychosocial changes that occur at different stages of development, caution should be exercised in generalizing these findings to other age groups.

Another limitation is that the data come from the turn of the century. Since then, research has indicated increases in loneliness, depressive symptoms, and related mental-health problems among young people (Cybulski et al., 2021; Duffy et al., 2019; Lien et al., 2023). The social contexts in which children and adolescents establish, maintain, and evaluate peer relationships have also changed substantially with the development of digital communication platforms (Nesi et al., 2018a, 2018b). Contemporary online environments may introduce additional interpersonal mechanisms relevant to loneliness–depression dynamics, including social comparison and feedback-seeking (Nesi & Prinstein, 2015), cybervictimization (Hu et al., 2021), digitally amplified peer-status processes such as visible exclusion and quantifiable peer feedback (Nesi et al., 2018a, 2018b), and sleep disruption related to intensive or late-night digital media use (Odgers & Jensen, 2020; Scott et al., 2019). These mechanisms may strengthen, weaken, or reshape some of the prospective pathways observed in the present study. At the same time, digital communication should not be interpreted as uniformly harmful, as it may also provide companionship, emotional support, and access to peer communities (Antheunis et al., 2016). Reviews and large-scale studies suggest that associations between digital technology use and adolescent mental health are heterogeneous and often small, rather than consistently negative (Odgers & Jensen, 2020; Orben & Przybylski, 2019). Thus, although the core affective and interpersonal processes identified in the present study may remain relevant, their strength, expression, and moderators should be re-examined in contemporary cohorts.

## 5. Conclusions

This study examined prospective symptom-level associations between loneliness and depression-related affective and behavioral states in late childhood using a cross-lagged panel network model. The findings show that loneliness is involved in both reciprocal and unidirectional longitudinal associations. Reciprocal associations were observed between loneliness and sadness, and between loneliness and perceived parental pressure. These results suggest that loneliness in late childhood may be embedded in mutually reinforcing affective and family-related interpersonal processes: loneliness may be followed by greater sadness and stronger perceptions of parental pressure, while sadness and perceived parental pressure may also contribute to later loneliness.

The findings also highlight several unidirectional associations. Higher happiness predicted lower subsequent loneliness, suggesting that positive affective states may play a protective role in relation to later loneliness. In contrast, higher boredom predicted higher subsequent loneliness, indicating that difficulties in engaging with meaningful or satisfying activities may be relevant to the development of loneliness over time. Loneliness, in turn, predicted later nervousness/tension and busyness. These latter associations suggest that loneliness may be followed by broader emotional distress and subjective overload, although the mechanisms underlying these links require further investigation.

Overall, the study indicates that loneliness should not be treated only as a correlate of general depressive symptom severity. Rather, it appears to be a specific component of a broader network of affective, behavioral, and interpersonal experiences. At the same time, the findings should be interpreted as prospective associations rather than evidence of causal mechanisms. Future longitudinal network studies should use standardized multi-item measures, additional measurement occasions, and more detailed assessments of family context, children’s activities, social withdrawal, relational appraisal, and emotion regulation. Such studies would help clarify how loneliness and depression-related states influence one another over time and which processes may be most relevant for developmentally sensitive prevention and intervention.

## Data Availability

The data for analysis were downloaded from https://www.nlsinfo.org/investigator/pages/login.jsp?p=timeout (accessed on 15 March 2024).

## Supplementary Materials

The following supporting information can be downloaded at: https://www.mdpi.com/article/10.3390/ejihpe16060078/s1, Table S1: Characteristics of studies on loneliness and network of symptoms of depression; Table S2: Descriptive statistics at baseline and follow-up; Table S3: Matrix of Spearman’s rho correlations between the study variables; Figure S1: Stability of edge centrality in the longitudinal cross-lagged network; Figure S2: Stability of out-strength and in-strength centrality in the longitudinal cross-lagged network; Figure S3: Autoregressive stability indices of variables between T1 and T2; Figure S4: Bootstrapped difference tests for cross-lagged edge weights (T1→T2); Figure S5: Accuracy of cross-lagged edge weights (T1→T2) with confidence intervals.

## Abbreviations

The following abbreviations are used in this manuscript:

UCLA	University of California, Los Angeles (UCLA) Loneliness Scale
DJGLS	De Jong Gierveld Loneliness Scale
NLSY79	Children and Young Adults survey
CBT	Cognitive-behavioral therapy
IPT	Interpersonal Therapy
CLPM	Cross-lagged panel modelling
CLPN	Cross-lagged panel network
