# Supplementary material for: The Longitudinal Interplay Between Loneliness and Depressive Symptoms During Late Childhood: Cross-Lagged Panel Network Analyses
Source: Eur J Investig Health Psychol Educ. 2026 May 31;16(6):78. doi: 10.3390/ejihpe16060078 (PMC13298037; doi:10.3390/ejihpe16060078)
Supplement: Supplementary file 1 [file ejihpe-16-00078-s001.zip › ejihpe-4214703-supplementary.pdf]

Table S1. Characteristics of studies on loneliness and network of symptoms of depression

A<br>Study	B<br>Depression questionnaire	C<br>Number of scale items	E<br>Mean age of respondents and/or age range	F<br>Sample size	G<br>Country	H<br>Type of population	I<br>Centrality rank of the loneliness	J<br>High correlation of loneliness with …
1. (Mullarkey et al., 2019)	CDI	26 items[A]	14.35 ± 0.66<br>13–19 years	1,409	USA	General	2	Sadness; Lack of friendship; Feeling unloved
2. (D. Kim et al., 2021)	CDI	27 items	6–12 years	10,233	Korea	General	1	Sadness; Crying; Lack of friendship; Suicidality; Somatic concern
3. (K. M. Kim et al., 2021)	CDI	27 items	14.21<br>(SD = 2.30)<br>7–19 years	455	Korea	Specific[1]	13	Sadness; Crying; Suicidality
4. (K. Li et al., 2021)	CDI	27 items	7–17 years	2,517	China	Specific[2]	5	Lack of friendship; Crying; Sleep disturbance
5. (Xie et al., 2022)	CDI	27 items	14.70 ± 1.72	571	China	General	2	Lack of friendship; Sadness; Suicidality; Crying
6. (Gijzen et al., 2021)	CDI-2	28 items	11–16 years	5,888	Netherlands	General	1	Lack of friendship; Feeling unimportant to family; Sadness; Feeling unloved; Suicidality
7. (Xie et al., 2022)	SMFQ	13 items	14.33 ± 3.60	2,194	China	General	6	Feeling unloved; Restless; Crying; Sadness
8. (Mullarkey et al., 2021)	SMFQ	13 items	14.72 ± 1.79<br>11–19 years	1,059	USA	General	4	Feeling unloved; Crying; Bad person; Sadness
9. (Manfro et al., 2021)	MFQ	33 items	14–16 years	7,772	Brazil	General	2	Feeling unloved; Not enjoy; Not want to see friends
10. (B. Kenny et al., 2021)	CES-D-10	10 items	12.59<br>(SD = 0.39)<br>10–15 years	4,421	Canada	General	2	Depressed; Low energy; Not happy
11. (Grygiel et al., 2023)	RCADS-MDD	10 items[B]	(SD = 0.55)<br>11.61-12.56	496	Poland	General	11	Worthlessness, Sadness, Anhedonia
12. (Gossage et al., 2022)	CDI-S	10 items	17 (SD = 0.35)	561	New Zealand	General	1	Lack of friendship; Sadness; Belief that will not work out

A<br>Study	B<br>Depression question-naire	C<br>Number of scale items	E<br>Mean age of respondents and/or age range	F<br>Sample size	G<br>Country	H<br>Type of population	I<br>Centrality rank of the loneliness	J<br>High correlation of loneliness with …
13. (Schlechter et al., 2023)[X]	SMFQ	13 items	12.95<br>(SD = 1.41)<br>10–15 years	4,235	UK	General	5	Feeling unloved; Not as good as other kids
14. (Schlechter et al., 2023)[Y]	SMFQ	13 items	13.77<br>(SD = 0.45)<br>11–17 years	11,176	UK	General	3	Feeling unloved; Not as good as other kids; Miserable or unhappy; I hated myself
15. (Schlechter et al., 2023)[Z]	SMFQ	13 items	11.30<br>(SD = 3.4)<br>11–17 years	643	UK	General	4	Feeling unloved; Tired; I hated myself
16. (Ramos-Vera et al., 2023)	RADS-2	30 items	15.24<br>(SD = 1.02)<br>13–18 years	917	Peru	General	14	Sadness; Social isolation; Not appreci-ated; Devalued by parents; Crying

Note. CDI = Children's Depression Inventory. CDI-2 = Children's Depression Inventory; CDI-S = Children's Depression Inventory; SMFQ = Short Mood and Feelings Questionnaire. MFQ = Mood and Feelings Questionnaire. CES-D-10 = Short form of the Center for Epidemiologic Studies Depression Scale. RADS-2 = Reynolds Adolescent Depression Scale (version 2). [A] = item related to Suicidality was omitted. [B] = The RCADS-MDD scale does not have an item that addresses loneliness. To measure it, both direct and indirect measures of loneliness from sources outside the RCADS scale were used. This explains why loneliness ranks 11th in centrality within the analyzed network, despite the RCADS-MDD scale measuring only 10 symptoms of depression. [X] = Mental Health of Children and Young People (MHCYP survey); [Y] = UK Millennium cohort study (MCS survey); [Z] = LAC survey; [1] = Clients of the child and adolescent psychiatry clinic; [2] = Left-behind children.

Table S2. Descriptive statistics at baseline and follow-up

Variables	Time 1					Time 2					Difference between Time 1 and Time 2				
	Hardly Ever	Some-times	Often	Mean	*SD*	Hardly Ever	Some-times	Often	Mean	*SD*	Paired *t* test			Marginal homogeneity test	
	Percent					Percent					*t*	*p*	Cohen's *d*	*Sd MH*	*p*
Sad and blue	51.1	41.6	7.3	1.56	.63	53.7	38.9	7.5	1.54	.63	2.03	.042	.031	2.03	.042
Nervous, tense or on edge	43.8	46.0	10.2	1.66	.65	43.8	46.2	10.0	1.66	.65	.181	.856	.003	.18	.856
Happy	3.8	20.6	75.7	2.72	.53	3.1	23.4	73.5	2.70	.52	1.50	.134	.023	1.50	.134
Bored	12.3	53.7	34.0	2.22	.65	10.4	53.4	36.2	2.26	.63	-3.47	<.001	-.053	-3.46	<.001
Lonely	51.7	34.5	13.8	1.62	.71	55.3	33.2	11.5	1.56	.69	4.53	<.001	.069	4.52	<.000
Tired or worn out	24.5	52.5	23.0	1.98	.69	21.9	56.6	21.5	2.00	.66	-.87	.384	-.013	-.87	.384
Excited about something that you ' re looking forward to	5.6	28.2	66.2	2.61	.59	4.8	31.0	64.2	2.59	.58	.97	.334	.015	.97	.334
Too busy to get everything done	33.6	47.9	18.5	1.85	.71	31.8	49.7	18.5	1.87	.70	-1.33	.184	-.020	-1.33	.184
Pressured by your mother or father	58.6	28.5	12.8	1.54	.71	59.8	29.0	11.2	1.51	.69	2.01	.045	.031	2.01	.045

Note. *SD* = Standard Deviation; *Sd MH* = Standardized MH statistics; *p* = *p* value.

Table S3. Matrix of rho Spearman correlations between the studied variables

Variable	1	2	3	4	5	6	7	8	9	10	11	12	13	14	15	16	17
Sad T1	--																
Nervous T1	.30**	--															
Happy T1	-.22**	-.12**	--														
Bored T1	.15**	.17**	-.07**	--													
Lonely T1	.31**	.22**	-.19**	.27**	--												
Tired T1	.14**	.19**	0.00	.19**	.15**	--											
Excited T1	-.06**	.04**	.29**	0.02	-.08**	.06**	--										
Busy T1	.16**	.19**	-.04**	.15**	.18**	.22**	.05**	--									
Pressured T1	.25**	.23**	-.17**	.18**	.31**	.16**	-.09**	.27**	--								
Sad T2	.25**	.14**	-.13**	.08**	.17**	.07**	-.06**	.09**	.11**	--							
Nervous T2	.15**	.18**	-.10**	.08**	.12**	.03*	-0.02	.06**	.11**	.37**	--						
Happy T2	-.14**	-.07**	.22**	-.08**	-.13**	-.04*	.11**	-.05**	-.15**	-.28**	-.16**	--					
Bored T2	.10**	.08**	-.08**	.27**	.11**	.10**	-0.02	.06**	.07**	.18**	.17**	-.10**	--				
Lonely T2	.18**	.11**	-.10**	.14**	.26**	.07**	-.06**	.09**	.16**	.39**	.25**	-.21**	.26**	--			
Tired T2	.05**	.08**	-.04*	.09**	.08**	.13**	-0.01	.07**	.08**	.14**	.20**	-0.03	.21**	.17**	--		
Excited T2	-.06**	0.00	.14**	-.05**	-.05**	-0.02	.14**	0.01	-.05**	-.13**	-.03*	.33**	-.04**	-.11**	0.01	--	
Busy T2	.07**	.07**	-0.02	0.03	.08**	.03*	0.02	.15**	.08**	.17**	.19**	-.05**	.12**	.19**	.22**	.03*	--
Pressured T2	.14**	.10**	-.10**	.08**	.17**	.06**	-.05**	.10**	.22**	.29**	.25**	-.20**	.13**	.27**	.15**	-.10**	.22**

Note. $^{*}p < 0.05$, $^{**}p < 0.01$.

European Journal of
Investigation in Health, Psychology and Education

Figure S1. Stability of edge centrality in longitudinal (cross-lagged) network

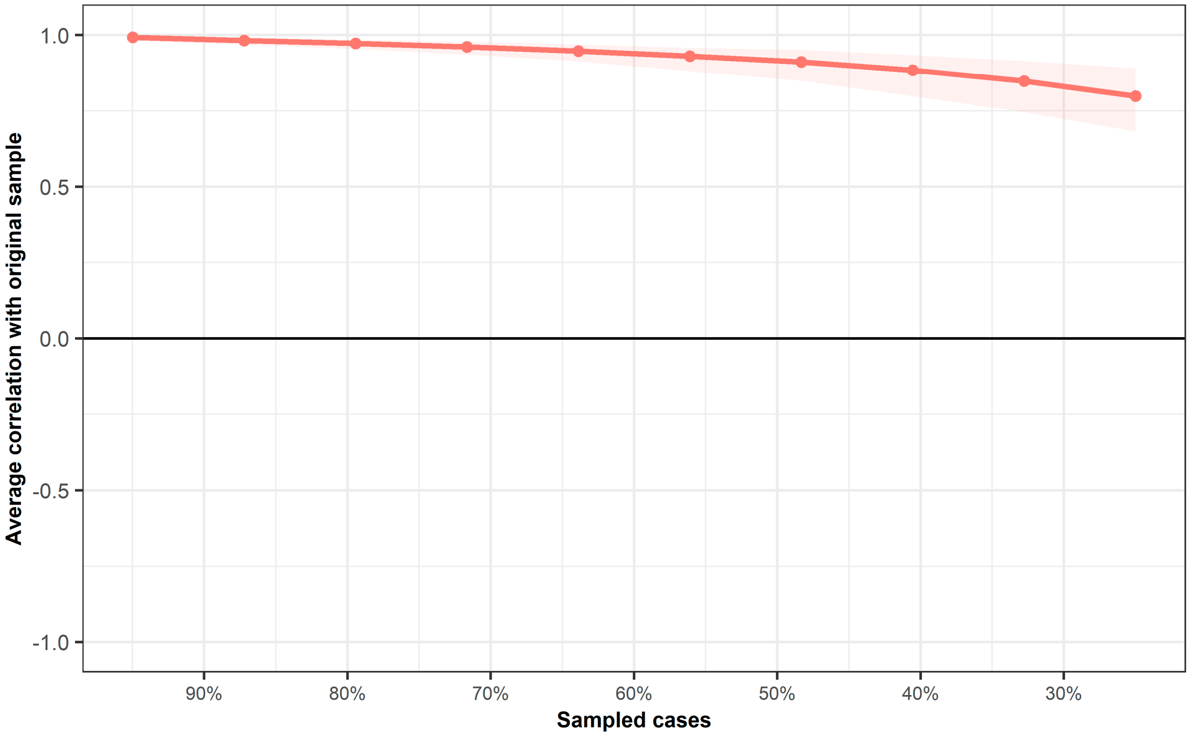

Figure S2. Stability of out-strength and in-strength centrality in longitudinal (cross-lagged) network

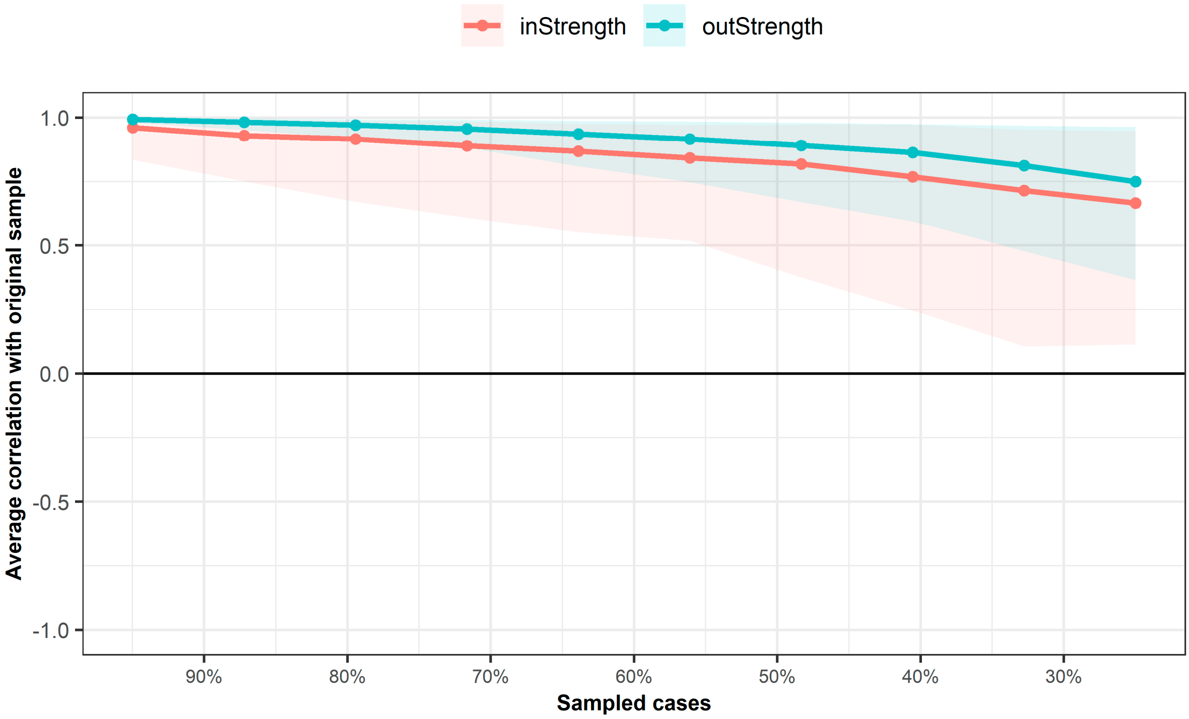

Figure S3. Indices of autoregression (stability) of variables between T1 and T2

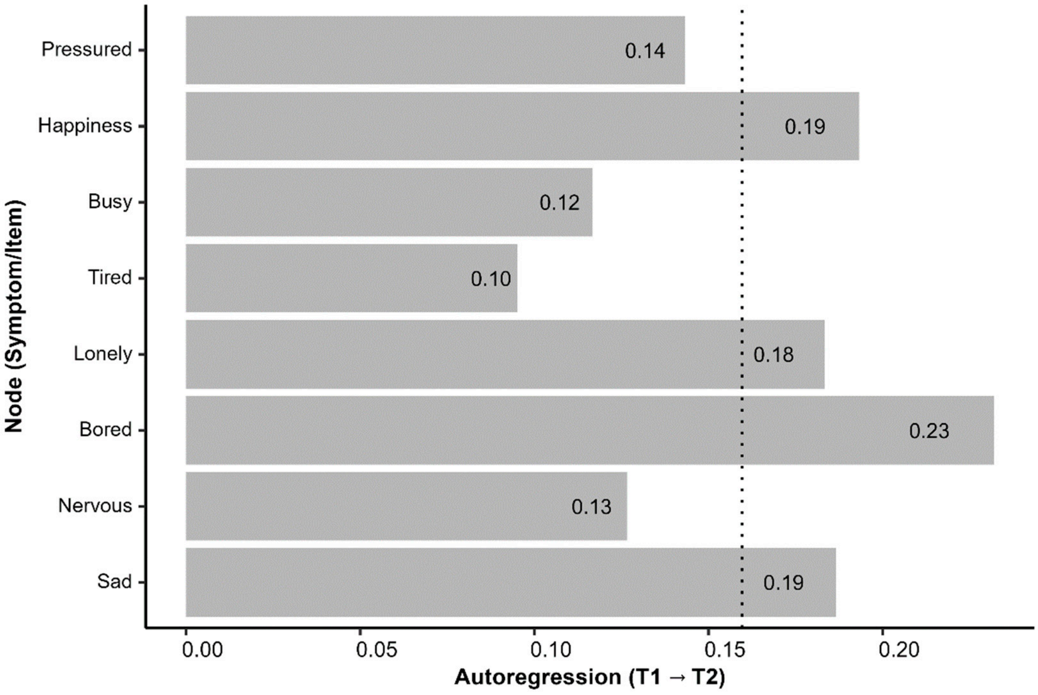

Figure S4. Plot of the bootstrapped difference tests (*α* = .05) for cross-lagged edge-weights (T1→T2)

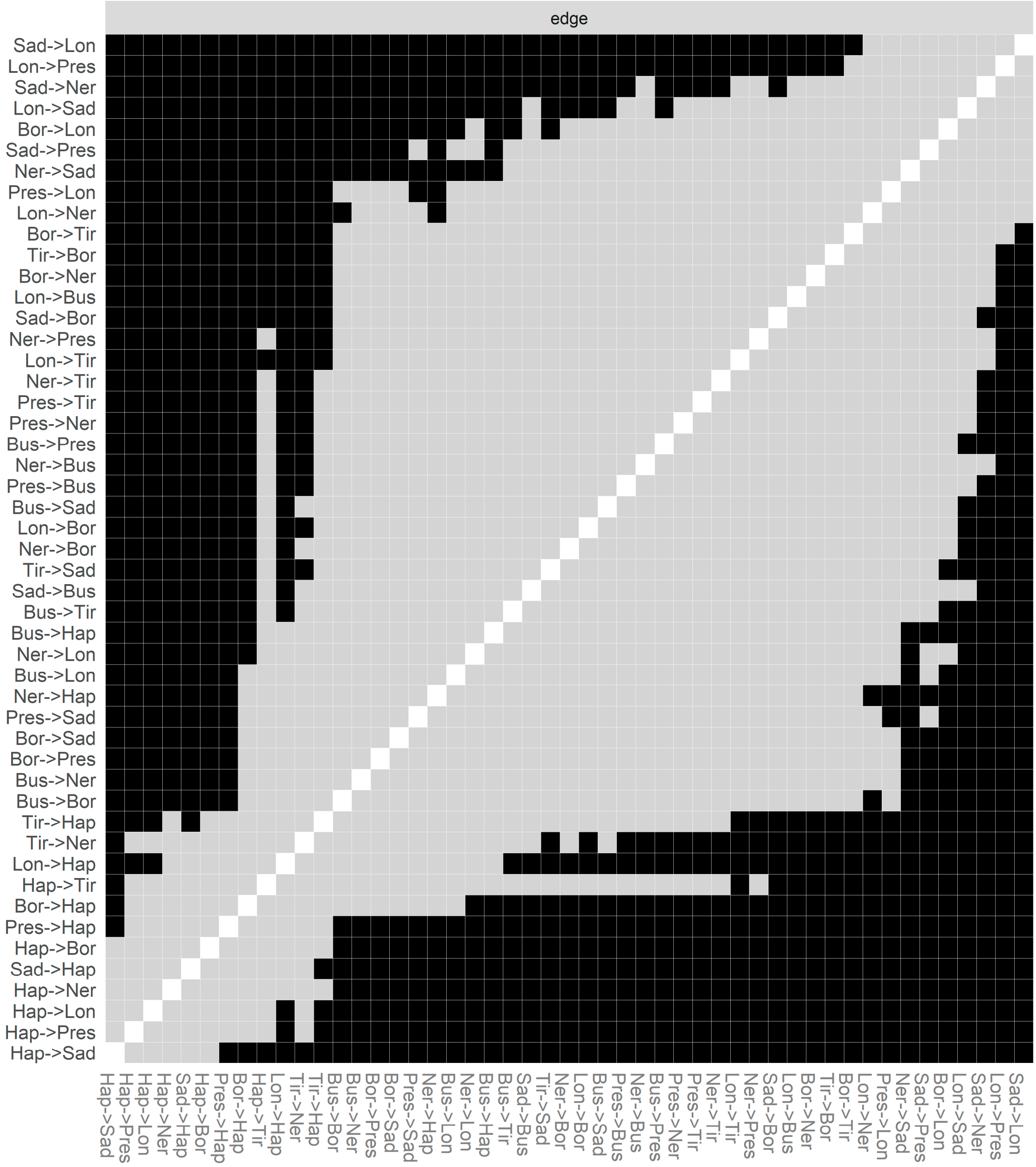

Note. Gray colored boxes represent nonsignificant differences, while black boxes represent significant differences

Figure S5. Accuracy of cross-lagged edge weights (T1→T2) with confidence intervals

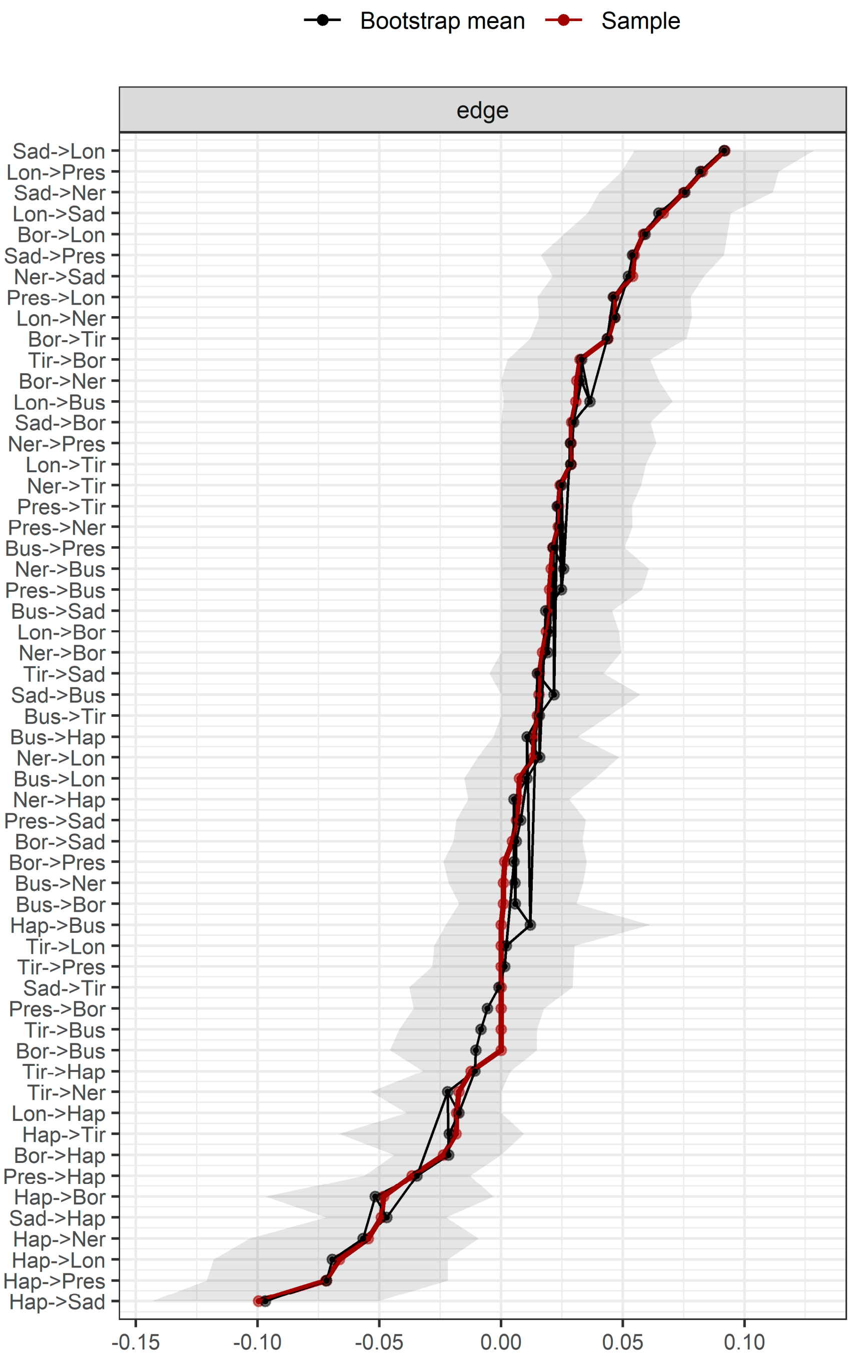

Note. Figure show edge estimate in the sample, the mean edge estimate in the bootstrapped samples and the 95% confidence interval band from the bootstraps edge weights.
